# Increasing Yield and Antioxidative Performance of Litchi Pericarp Procyanidins in Baked Food by Ultrasound-Assisted Extraction Coupled with Enzymatic Treatment

**DOI:** 10.3390/molecules23092089

**Published:** 2018-08-21

**Authors:** Shuyi Li, Yanjie Yang, Junsheng Li, Zhenzhou Zhu, Jose M. Lorenzo, Francisco J. Barba

**Affiliations:** 1 College of Food Science and Engineering, Wuhan Polytechnic University, Wuhan 430023, China; lishuyisz@sina.com (S.L.); 16602720428@163.com (Y.Y.); m13207138007@163.com (J.L.); 2 Hubei Key Laboratory for Processing and Transformation of Agricultural Products, Wuhan Polytechnic University, Wuhan 430023, China; 3 Centro Tecnológico de la Carne de Galicia, Adva. Galicia No. 4, Parque Tecnológico de Galicia, San Cibrao das Viñas, 32900 Ourense, Spain; jmlorenzo@ceteca.net; 4 Nutrition and Food Science Area, Preventive Medicine and Public Health, Food Science, Toxicology and Forensic Medicine Department, Faculty of Pharmacy, Universitat de València, Avda. Vicent Andrés Estellés, s/n, Burjassot, 46100 València, Spain

**Keywords:** litchi pericarp, oligomeric procyanidins, RSM (Response Surface Methodology), HPLC, baked food

## Abstract

Extraction with organic solvents is a traditional method to isolate bioactive compounds, which is energy-wasting and time-consuming. Therefore, enzyme and ultrasound treatments were combined to assist the extraction of oligomeric procyanidins from litchi pericarp (LPOPC), as an innovative approach to replace conventional extraction methods. Under optimum conditions (enzyme concentration 0.12 mg/mL, ultrasonic power 300 W, ultrasonic time 80 min, and liquid/solid ratio 10 mL/g), the yield of LPOPC could be improved up to 13.5%. HPLC analysis indicated that the oligomeric procyanidins (OPC) content of LPOPC from proposed extraction was up to 89.6%, mainly including (−)-epicatechin, procyanidin A1, A2, and A-type procyanidin trimer. Moreover, LPOPC powder was added in baked food to inhibit the lipid peroxidation. It was found that 0.2% (*w/w*) of LPOPC could maintain the quality of cookies in the first 7 days, by decreasing the peroxide values. The procyanidin dimers and trimers in LPOPC played more important roles as antioxidants compared to monomers during storage. The results also showed that the combined extraction process can be considered as a useful and efficient method for the extraction of functional components from other plant sources.

## 1. Introduction

**Figure 1:**
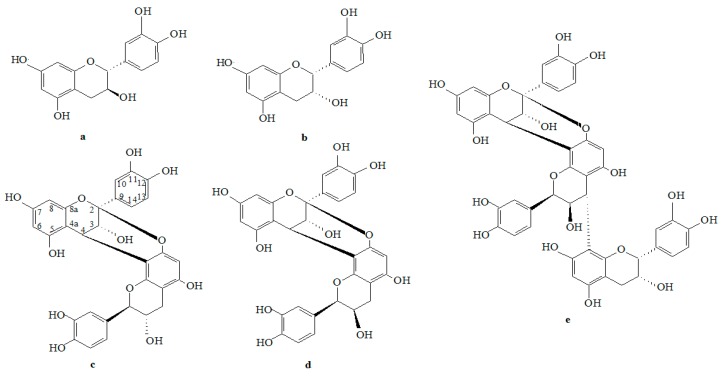
Structures of CC, EC, A1, A2, and A-3 in litchi pericarp (**a**–**e**, respectively).

Litchi (*Litchi chinensis* Sonn.) is a subtropical fruit with high commercial value, which is widely cultivated in the south of China. Besides the delicious fruit, litchi flowers, seeds, and pericarp also exhibit important biological activities [1,2,3]. As it was previously reported, litchi pericarp, which represents ≈15% of fresh fruit, is rich in oligomeric procyanidins (OPC), especially A-type, associated with anthocyanidins and other polyphenols, making the bright color of the peel [4,5]. Intriguingly, although the crude extraction rate of procyanidins from litchi pericarp is ≈2%, the relative content of procyanidins is greater than 95%, mainly composed of (−)-epicatechin (EC), (+)-catechin (CC), procyanidin A1, epicatechin-(4β→8, 2β→O→7)-catechin, procyanidin A2, epicatechin-(4β→8, 2β→O→7)-epicatechin, B2, epicatechin-4β→8-epicatechin, A-type procyanidin trimer (A-3), epicatechin-(4β→8, 2β→O→7)-epicatechin-(4β→8)-epicatechin (Figure 1), and their oligomeric isomers [2,5].

Procyanidins are a general class of polyphenolic compounds based on flavan-3-ol unit, which can be widely found in the plant kingdom, and they are used as a functional dietary supplement [6,7,8,9]. Previous researches have demonstrated that OPC from litchi pericarp (LPOPC) can facilitate scavenging oxygen free radicals, inhibiting lipid peroxidation and chelating metal ions, for further effectiveness on anti-atherosclerosis, anti-cancer and anti-diabetes [10,11,12]. For instance, in recent years, Japanese researchers have developed lychee polyphenols-rich (mainly proanthocyanidins monomer and oligomer) health care products known as Oligonol^®^, even suggesting its ability to inhibit and treat hyperuricemia and gout conditions [13]. However, less attention has been brought to the application of LPOPC in processed food, and there is not a clear consensus regarding OPC antioxidant performance in baked food.

Nowadays, the extraction methods of proanthocyanidins mainly referred to organic solvent extraction, ultrasound-assisted extraction and enzymatic hydrolysis [14,15]. For the organic solvent extraction method, the overuse of the chemicals prolongs the recovery process which is time-consuming and contaminates the environment. Ultrasonic extraction (UAE) has the advantages of fast, safe, energy-saving, and high extraction rate, while enzymatic hydrolysis is green and environmentally friendly, which is also easy to manage [16].

In this study, we applied the response surface methodology (RSM) to optimize enzymatic treatment and ultrasound-assisted extraction for obtaining procyanidins from litchi pericarp, in which enzymatic treatment was combined with ultrasound extraction. In addition, HPLC analysis and peroxide value examination was carried out to demonstrate the antioxidant effect of LPOPC, included in the extract, added in cookies, thus illustrating how procyanidins affect the storage properties of high-fat foods.

## 2. Results and Discussion

### 2.1. Effect of Combined Enzyme/Ultrasonic Treatment on the Yield of LPOPC

It is known that increased bioactives´ release from plant cells by cell disruption and extraction through the cell wall can be optimized using enzyme mixtures [17]. In present study, it was also evidenced that combining pectinase and cellulose could promote the isolation of procyanidins. The yield rate of LPOPC after enzymatic treatment could be ≈7.5%, which to some extent facilitates the preparation. To further investigate the effect of ultrasound conditions on the release of procyanidins from litchi pericarp, different tests using RSM were conducted. The parameters taken into account were ultrasound power, ultrasound time, and liquid-to-material ratio. The response was yield of LPOPC. Prior to the ultrasonic treatment, enzyme treatment was carried out according to Section 3.3. As can be seen in Table 1, yield of LPOPC obtained after combined enzyme/ultrasound-assisted extraction (UAE) process varied from 8.25% to 14.20%. Compared with the process with only enzymatic treatment, which led to the LPOPC yield less than 9%, the advantage of combined process was significant.

**Figure 2:**
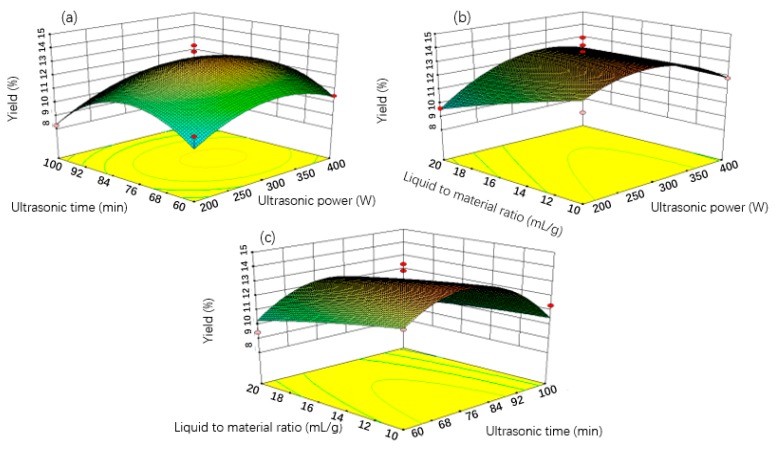
Response surface plots showing the operating parameter effects on extraction yield: (**a**) Ultrasonic time vs. ultrasonic power at fixed liquid-to-material ratio of 15 mL/g; (**b**) liquid-to-material ratio vs. ultrasonic power at fixed ultrasonic time at 80 min; (**c**) liquid-to-material ratio vs. ultrasonic time at fixed ultrasonic power of 300 W.

To have a better visualization of the factors derived from the statistical analysis, three-dimensional (3D), response surface plots and contour plots representing the effects of the independent variables on OPC yield are shown in Figure 2. The factors involving ultrasonic power, extraction time and ratio of liquid to material all affected extraction efficiency. 

The yield rate of OPC increased quickly and reached a maximum level at 300 W, after that, the values declined gradually, which indicated that the cavitation effect resulting from ultrasonic power on the isolation of polyphenols was bidirectional, both positive and negative. Meanwhile, increasing ultrasonic power promoted an increased temperature, thus promoting OPC degradation [18]. Thus, 300 W is considered to be the optimal intensity of ultrasonic power corresponding to the purpose of saving energy and reducing the degradation of thermolabile compounds [19]. The effect of ultrasonic time and liquid-to-material ratio also showed a similar trend, thus facilitating OPC extraction when both were increased and then, after reaching a plateau, decreasing OPC extraction. These results can be related to the increasing ultrasonic period, which leads to the further disruption of material cell and speeds up both the release and diffusion of the procyanidins into solvent [14]. However, an excessive extension in the processing time will trigger oxidization or degradation of procyanidins, lowering the yield quality of OPC. A greater ratio of liquid to material indicates higher concentration difference between the interior plant cells and the exterior solvent, and the diffusion of OPC occurred more quickly, which could lead to enhancement of efficiency [20]. However, the yield value of OPC decreased slowly once the ratio met the point at 20 mL/g. When the ratios increased markedly, the ultrasonic energy attached to the unit volume would fall, resulting in the decrease of OPC yield [21].

The ANOVA for fitted quadratic polynomial model of extraction of procyanidins was presented in Table 2. The statistical significance of the model equations was evaluated by the *F*-test and *p*-value, which suggested that the regression model is significant. With the analysis of variance (ANOVA), the final equation predicting the behavior of the extraction system is provided by the following Equation (1):

(1) $$\mathrm{Yield}\;(\%)=13.15+0.46X_{1}-0.59X_{2}-0.69X_{3}-0.075X_{1}X_{2}+0.84X_{1}X_{3}+0.26X_{2}X_{3}-1.48X_{1}^{2}-2.38X_{2}^{2}-0.11X_{3}^{2}\;,$$

According to the above equation, the linear term of ultrasonic sound exhibited positive effects on OPC yield, while the other two parameters of the extraction conditions exhibited a negative impact. The predicted optimal extraction conditions for maximal extraction yield of OPC were as follows: ultrasonic power of 287 W, ultrasonic time of 76 min and the ratio of liquid to material at 10 mL/g. Under these conditions, the predicted highest yield of procyanidins was 13.83%. Based on the reliability conditions of experimental process, the available parameters for maximum extraction yield of OPC were updated to ultrasonic power 300 W, ultrasonic time 80 min, and the liquid-to-solid ratio of 10 mL/g. Referring to these optimal conditions, the actual maximum yield of OPC was 13.5%, which was in close agreement with the expected values, thus validating the suitability of the fitted RSM model. In other words, the final yield rate of OPC from litchi pericarp could be estimated to increase by more than six times depending on UAE coupled with enzymatic treatment, compared to that of traditional ethanol extracts [2,5,22].

### 2.2. Oligomericcomposition of Different Litchi Pericarp Procyanidins

**Figure 3:**
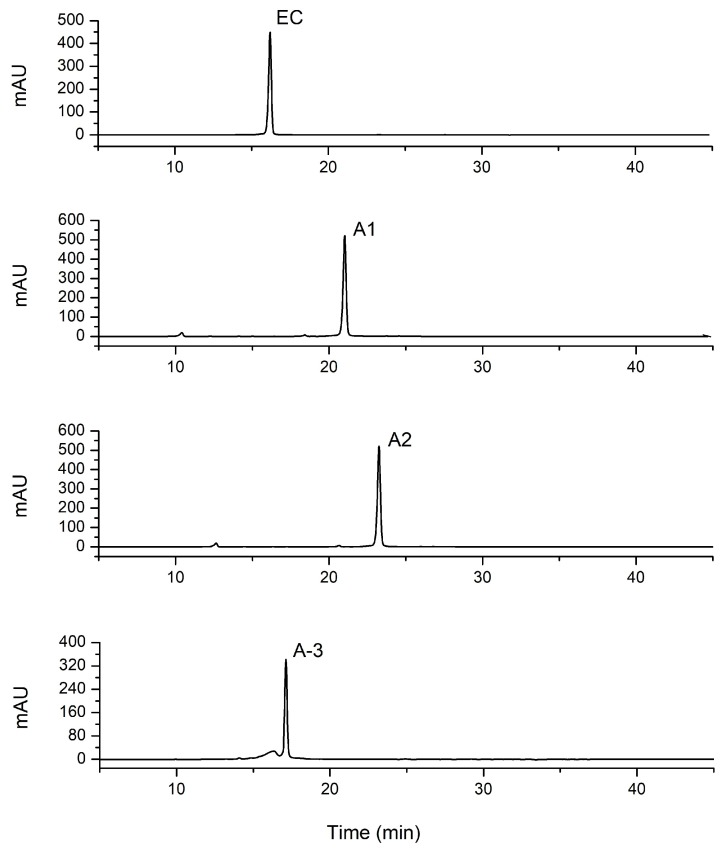
Specific chromatograms of EC, A1, A2, and A-3 standard by HPLC analysis.

**Figure 4:**
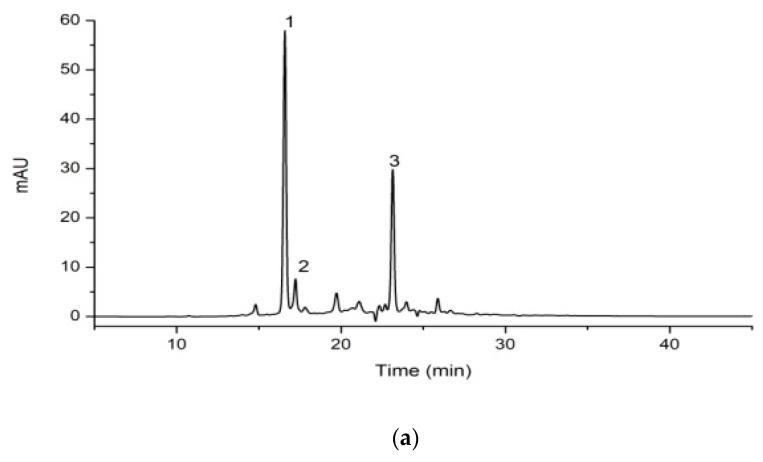

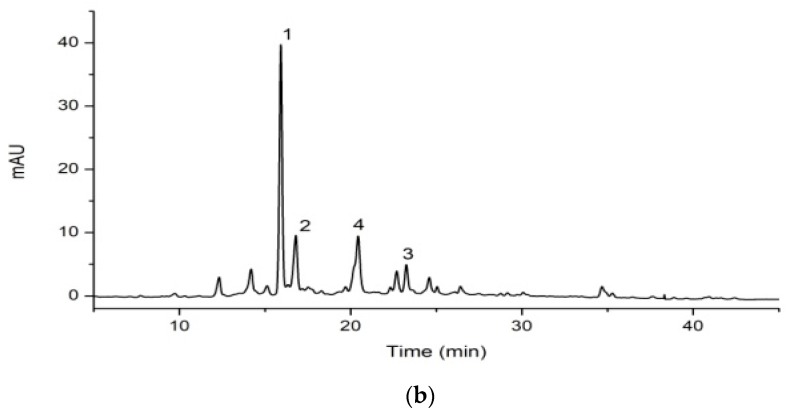
HPLC analysis of oligomeric procyanidins from litchi pericarp (LPOPC) from enzyme (**a**)—or ultrasound (**b**)—assisted extraction. Peaks 1–4 represented EC, A-3, A2, and A1, respectively.

As reported, the procyanidins from litchi pericarp identified were mainly (−)-epicatechin, A-type procyanidin dimer and trimer, accounting for more than 60% of the oligomeric extracts. The chromatogram of procyanidin standards, including EC, A1, A2, A3, purified from LPOPC is shown in Figure 3. The presence of procyanidin monomer (EC), A2, and A3, in all the LPOPC extracts from enzyme and/or ultrasound-assisted extraction process (corresponding to Peaks 1, 3, 2, respectively), was confirmed by comparing with the retention time and UV spectrum of standards. According to the HPLC analysis, it was obviously indicated that the composition of LPOPC extracted from different methods varied from enzymatic hydrolysis to ultrasound intensification (Figure 4). It can be also found that the relative contents of monomers and dimers were much higher before ultrasound treatment (Figure 4a). However, an isomer of A2 (Peak 4) was detected in LPOPC from ultrasound-assisted extraction (Figure 4b), which was inferred to be procyanidin A1, epicatechin-(4β→8, 2β→O→7)-catechin [23]. It has been evidenced by Li et al. [24] that procyanidin A2 could be transformed to A1 at certain conditions, possibly related to ultrasonic energy effect. Moreover, the proportion of A-type trimer was slightly increased by the intensified physical field (Figure 4b). Each component in LPOPC consists of mainly one flavanolic compound, in which the peak area and relative abundance of the component in the chromatogram represent its proportion [5]. After homogenization and calculation of the ratios of main peak area, it was shown that the total relative content of flavanols in LPOPC with enzyme induced was 94.0%, while the extract from further ultrasonic treatment displayed lower purity, containing 89.6% of flavanols, in which the procyanidin oligomers played a more dominant role than monomer.

### 2.3. Inhibitory Effect of LPOPC on Lipid Oxidation in Cookies

#### 2.3.1. Sensory Evaluation and Proper Addition of LPOPC

As can be depicted from the sensory evaluation (data not shown), it was indicated that once the proportion of LPOPC added is up to 0.3%, the shape of the biscuit is hard to be formed, when molding process turns difficult and slowly with a deep color, rough tissue and astringent taste. Sustaining the content of LPOPC around 0.2%, molding is easily accessible, and the color of cookies is adequated, while the taste is crisp and non-stick teeth with fragrance and a pleasant smell. After being kept at room temperature for 4 days, the peroxide values (PVs) of cookies with different concentrations of LPOPC (0, 0.1%, 0.2%, and 0.3%) were measured to be 70.12 ± 3.21, 64.34 ± 2.65, 50.74 ± 3.14, and 43.85 ± 1.91 meq/kg, respectively, illustrating the strong antioxidant activity of LPOPC in baked food. Based on the results from sensory evaluation and the PV, the optimum dosage of LPOPC was selected to be 0.2% for application in cookies.

We further monitored the lipid oxidation of cookies in 10 days (Table 3). It was found that the PV of food increased slowly at the first 7 days, but after this time, the fat in cookies was quickly oxidized. As it is well known, LPOPCs are natural oxygen free radical scavenger and lipid peroxidation inhibitors. Therefore, the rapid lipid oxidation and detrimental effects in quality observed in later periods was probably due to the change of LPOPC during high-temperature heating and long-term storage, including both transformation and degradation. 

#### 2.3.2. Changes of Procyanidins in Baked Food after Storage

**Figure 5:**
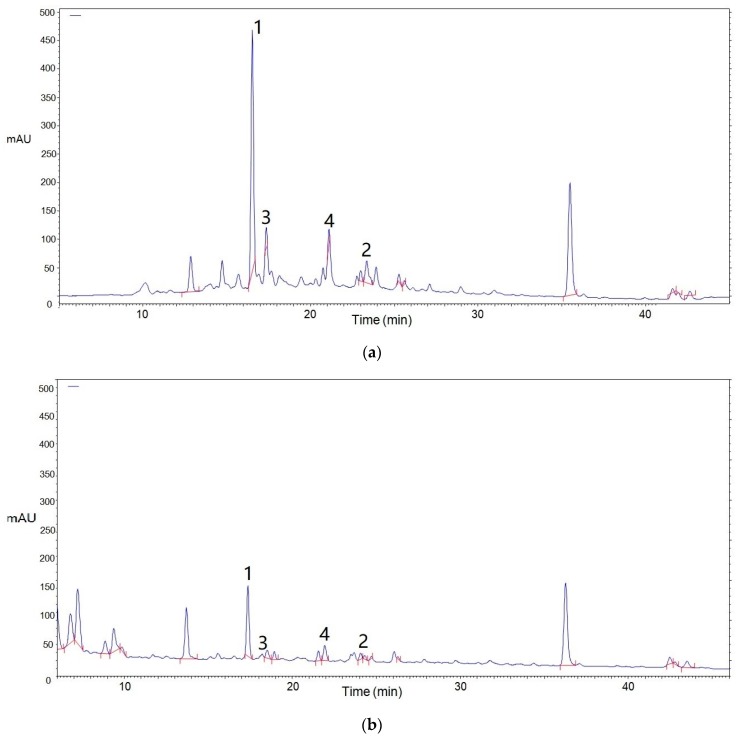
Composition of procyanidin oligomers in litchi pericarp before (**a**) and after (**b**) baked in cookies. Peaks 1–4 represented EC, A-3, A2, and A1, respectively.

In order to figure out the possible reasons of the loss of antioxidant capacity of LPOPC after storage, the composition of procyanidins in the cookies was analyzed. The comparable chromatograms are shown in Figure 5. As can be seen in the figure, the total content of procyanidins present in biscuits decreased significantly, in which procyanidin monomer (Peak 1) was found to be decreased nearly 70% compared to the original sample. The procyanidin dimer and trimer (Peaks 2–4) can be hardly detected, although less exhausted referring to the drop rate of peak area. These results are in agreement with the deduction that the depletion of procyanidins leads to promoting lipid oxidation. Procyanidins, as a free radical quencher, participate in the inhibition of lipid peroxidation process involving dehydrogenation, oxidation, cracking reaction. Procyanidin dimers and trimers are more important than monomer as antioxidants [25]. Namely, the oligomers in the LPOPC exhibited higher functional ability on inhibition of peroxidation of high-fat baked food. The results evidenced that the anti-liposomal oxidation of procyanidins is closely related to the degree of polymerization [26,27], and their antioxidant activity will be significantly decreased with the increase of polymerization degree [28], but its mechanism in food systems was not clearly unveiled. More knowledge associated with mass spectrum and NMR analysis on the transformation of food-related polyphenols is still needed.

## 3. Material and Methods

### 3.1. Chemicals and Materials

Fresh litchi fruits were collected from Guangzhou, Guangdong province of China, stored at −20 °C until use. Prior to extraction, the whole litchi rinds were cut into the size of a fingernail. The mixture of cellulase (Macklin^®^, 50,000 U/g) and pectinase (Macklin^®^, Ningbo, Shanghai, China, 30,000 U/g) was used to accelerate release of polyphenols. Grape seed procyanidins (GSPC, purity > 95%) was purchased from Tianjing Natural Product R&D Co., Ltd. (Tianjing, China), which was applied to identify the relative content of procyanidin of extract as a standard.

### 3.2. Determination of Procyanidin Content

Procyanidin content of extracts was measured using the butanol–HCl method [29]. A standard curve was established related to the absorbance of GSPC solutions at different concentrations: 500, 400, 300, 200, 100, and 50 μg/mL, from which a linear fitting equation was obtained. Referring to the traditional process, 1 mL of the standard/sample solution was taken, followed by adding 0.2 mL of ammonium ferric sulfate (2%) and 6 mL of *n*-butanol-hydrochloric acid solution (95:5, *v/v*). The mixture was shaken 60 s, then it was kept in boiling water for 40 min and rapidly cooled. At last, the resultant absorbance was measured at 546 nm, in order to determine the relative content and weight of procyanidins according to the linear equation [5].

### 3.3. Enzyme Treatment-Assisted Extraction of LPOPC

Five grams of shredded litchi pericarp were weighed and immersed in 20% of ethanol solution (liquid ratio 1:15, *w/v*) at 50 °C for enzymatic treatment with cellulose (CE) and pectinase (PE). According to previous tests, for the sake of optimal hydrolysis, the ratio of CE and PE was at 1:1 (*m/m*), and the mixed enzyme concentration was 0.12 mg/mL. The treatment duration was 90 min. After extraction, the extract was centrifuged at 4000 rpm for 15 min to remove the insoluble compounds. The supernatant was collected and concentrated to a certain volume under reduced pressure at 40 °C, and then freeze-dried to obtain crude LPOPC powder. The extraction yield of LPOPC was defined as follows:

(2) $$\mathrm{Yield}(\%)=\frac{\mathrm{weight}\;\mathrm{of}\;\mathrm{procyanidins}\;(g)}{\mathrm{weight}\;\mathrm{of}\;\mathrm{litchi}\;\mathrm{pericarp}\;(g)}\times 100\%,$$

### 3.4. Enzyme/Ultrasound-Assisted Extraction of LPOPC

After enzymatic treatment, as in Section 3.3, the UAE was performed in an ultrasound device (Ultrasonic, microwave, ultraviolet Trinity reactor, Ningbo Scientz Biotechnology Co. LTD, Zhejiang, China) equipped with a temperature controller and a digital timer. When all of the processes finished, the LPOPC extract was also isolated by centrifugation, concentration, and lyophilisation. Response surface methodology (RSM) was used to estimate and optimize the UAE process for extraction of procyanidins from litchi pericarp. As presented in Table 4, Ultrasound power (X_1_, W) ranging from 200 to 400 W, ultrasound time (X_2_, min) from 60 to 100 min and liquid-to-material ratio (X_3_, mL/g) from 10 to 20 mL/g were selected as independent variables. In order to normalize the parameters, each of the coded variables were ranged from −1 to 1, so that they all affect the response more evenly, and the units of the parameters were irrelevant [30]. The Box–Behnken Design (BBD) with three variables and three levels was constructed, when the yield values of OPC designated as the response values. Regression analysis was carried out on the experimental data from BBD, and the second-order polynomial model was performed as follows:

(3) $${Y=A}_{0}+\sum_{i=1}^{3}A_{i}X_{i}+\sum_{i=1}^{3}A_{\mathrm{ii}}X_{i}^{2}+\sum_{i=1}^{2}\sum_{j=i+1}^{3}A_{\mathrm{ij}}X_{i}X_{j},$$

where Y is the predicted response, A_0_ is a constant, A_i_ is linear coefficient, A_ii_ is quadratic coefficient and A_ij_ is interaction coefficient. X_i_ and X_j_ are independent variables.

### 3.5. Analysis of Oligomeric Composition of LPOPC by HPLC

Prior to analysis, the crude LPOPC extract was purified by using AB-8 resin (0.3–1.25 mm particle size, Nankai Hecheng Science & Technology Co., Tianjin, China), which was recovered in 70% ethanol [5]. The eluent was evaporated and the high-purity procyanidin extract was obtained. A couple of purified LPOPC extracts from a different extraction process were then analyzed, both dissolved in methanol at 0.5 mg/mL. The main flavanolic compounds in litchi pericarp, which were identified as EC, A1, A2, and A-3 previously, were individually prepared using the gel chromatographic methods previously described by Li et al. [5,21] and considered as standards to determine and quantify the relative content of each compound in LPOPC. The standard compounds (purity > 95%) were diluted to 1.0 mg/mL, respectively. After filtration through the 0.22 μm filter, the solution was injected into an HPLC system (Agilent Technologies Co., Ltd., Santa Clara, CA, USA). The Agilent 1100 Series HPLC is equipped with a binary gradient pump, a diode array (DAD) detector, a column oven and an auto sampler (Agilent Technologies Co. Ltd., Santa Clara, CA, USA). Chromatographic conditions were performed as follows: Column, A ZORBAX Eclipse XDB-C18 (150 mm × 4.6 mm, 5 μm); column temperature, 28 °C; mobile phase, 0.4% of acetic acid solution and acetonitrile; elution program as shown in Table 1; flow rate, 1 mL/min; detection wavelength, 280 nm; injection volume, 10 μL. The relative content of individual procyanidin could be obtained due to normalization analysis, when it is referred to the presented peak area of each compound in LPOPC and compared with standards. The comparable composition of the two LPOPC could be also illustrated by calculating the ratio of specific chromatographic peak based on peak area.

### 3.6. Determination of LPOPC on Lipid Peroxidation in Baked Food

#### 3.6.1. Manual Preparation of Cookies

After stirring softened butter (approximate 65 g) to fluffy, 25 g of sugar and corn flour were added in the mud, individually. Continuing to stir the mixture till fully soft, 28 g of egg yolk were added into the pasta and blended to completely mixed, which was followed by introducing 100 g of low-gluten flour at presence of 0.1%, 0.2%, and 0.3% of LPOPC (*w*/*w*, purified extract from Section 3.4), respectively. The further shaped cookies were baked in an oven at 150 °C for 30 min, after which they were taken out and cooled down prior to analysis.

#### 3.6.2. Determination of the Optimum Addition of LPOPC

The optimal addition of procyanidin in cookies is based on the properties of the raw material and the acceptability of the final product, i.e., whether the sensory properties of the product are acceptable or attractive to consumers. Twenty people were invited to taste biscuits with different concentrations of LPOPC, making judgment on their integrity, frangibility, flavour, and color to primarily select the proper polyphenol dose introduced (data not shown).

Furthermore, all cookies were stored at room temperature for 4 days. The peroxide value (PV) of food was determined by iodometric method after the crude oil of cookies was isolated using Soxhlet organic reagents [21]. As a result, PVs can be combined with the sensory evaluation results to finally ensure the optimum dosage of procyanidins. 

#### 3.6.3. Evaluation on Changes of LPOPC After Storage

LPOPC was added to the raw cookies at the optimum mass ratio and stored at room temperature for 10 days since preparation. The remaining litchi procyanidins in biscuit crumbs were re-dissolved in chromatographic methanol. After further centrifugation and filtration, the changeable composition of LPOPC in the extract was determined by HPLC (same conditions as Section 3.5), using original procyanidin samples at considerably equal content (5 mg/mL) as control.

### 3.7. Statistical Analysis

All the experiments were repeated at least three times, and mean values and standard deviations were calculated. The values of error bars in the figures are equal to mean standard deviations (means ± SD). The significance of difference between groups was determined using an analysis of variance (ANOVA) by means of the software Origin Pro 8.0 (Northampton, MA, USA).

## 4. Conclusions

In the present study, the combined enzymatic and ultrasound treatment was used to intensify extraction of oligomeric procyanidins from litchi pericarp (LPOPC). Optimized ultrasonic treatment parameters were obtained by RSM experimental design. Based on the optimal extraction conditions, the extraction yield of LPOPC can be up to 13.5%, which is six times higher than that obtained after traditional ethanol extraction. Although the relative procyanidin content of LPOPC from combined extraction process was 89.6%, slightly lower than that from enzyme-assisted extraction, it showed more abundance in the variety of oligomers, suggesting that ultrasonic efforts can give birth to the transformation of flavanol compounds during extraction. In other words, the extraction technology involving ultrasound treatment has the potential to be scaled-up and be commonly used in the production of procyanidins from plant resources. However, the conditions related to the industrialization level should be different, therefore necessitating furthers studies for optimizing processing conditions. At the same time, 0.2% of LPOPC in processed cookies indicated strong antioxidant activity, in particular, anti-liposome oxidation ability. It was found that A-type procyanidin dimer and trimer played a more important role than the monomer in the inhibition of peroxidation. However, with the consumption of procyanidins in the heating and storage process of baked food, the antioxidant capacity of LPOPC was weakened, indicating that the direct addition of antioxidants in baked food cannot be for a long time. This study also shows that the coupled extraction technology can be regarded as a new type of green, simple, rapid, and efficient method for the extraction of bioactive components. 

## Figures and Tables

**Table 1:** Box–Behnken design for independent variables and observed responses of oligomeric procyanidins (OPC) from ultrasonic extraction.

Run (No.)	Ultrasound Power (W)	Ultrasound Time (min)	Liquid-to-Solid Ratio (mL/g)	Yield (%)
1	300 (0	60 (−1)	20 (1)	9.45
2	400 (1)	80 (0)	10 (−1)	11.85
3	200 (−1)	80 (0)	20 (1)	9.60
4	300 (0)	80 (0)	15 (0)	13.75
5	300 (0)	80 (0)	15 (0)	13.05
6	200 (−1)	100 (1)	15 (0)	8.25
7	400 (1)	80 (0)	20 (1)	12.95
8	200 (−1)	80 (0)	10 (−1)	11.85
9	300 (0)	80 (0)	15 (0)	12.45
10	200 (−1)	60 (−1)	15 (0)	10.20
11	300 (0)	80 (1)	15 (0)	12.30
12	300 (0)	60 (−1)	10 (−1)	12.15
13	300 (0)	100 (1)	20 (1)	9.70
14	300 (0)	100 (1)	10 (−1)	11.35
15	300 (0)	80 (0)	15 (0)	14.20
16	400 (1)	100 (1)	15 (0)	8.25
17	400 (1)	60 (−1)	15 (0)	10.50

**Table 2:** Analysis of variance (ANOVA) of response surface quadratic model analysis for the ultrasonic extraction.

Source	df	SS	MS	*F*-Value	Prob > F	Significant
X_1_	1	1.67	1.67	1.72	0.2316	
X_2_	1	2.82	2.81	2.91	0.1320	
X_3_	1	3.78	3.78	3.90	0.0890	
X_1_ × X_2_	1	0.023	0.023	0.023	0.8833	
X_1_ × X_3_	1	2.81	2.81	2.89	0.1329	
X_2_ × X_3_	1	0.28	0.28	0.28	0.6106	
X_12_	1	9.16	9.16	9.44	0.0180	
X_22_	1	23.75	23.75	24.47	0.0017	
X_32_	1	0.053	0.053	0.055	0.8214	
Models	9	46.38	5.15	5.31	0.0193	Significant
Residual	7	6.79	0.97			
Lack of fit	3	4.11	1.37	2.04	0.2509	Not Significant
Pure error	4	2.68	0.67			
Cor Total	16	53.17				
Adeq Precision	6.386					
C.V. (%)	8.73					

**Table 3:** Effect of storage time on the peroxide value (PV) of cookies with oligomeric procyanidins from litchi pericarp (LPOPC).

No.	Storage Time (d)	PV (meq/Kg)
1	4	50.45 ± 2.14
2	7	68.21 ± 1.85
3	10	113.94 ± 4.39

**Table 4:** Independent factor and their levels used in ultrasound-assisted extraction of procyanidins.

Factors	Levels		
	−1	0	1
Ultrasound Power (X_1_)/W	200	300	400
Ultrasound Time (X_2_)/min	60	80	100
Liquid-to-material ratio (X_3_)/mL/g	10	15	20
